# The Resilience of Polish Doctors and Their Behavioral Patterns in Coping with Work-Related Stress

**DOI:** 10.3390/jcm13247539

**Published:** 2024-12-11

**Authors:** Sławomir Wojczyk, Józefa Dąbek, Oskar Sierka, Tomasz Gąsior

**Affiliations:** 1 Department of Vascular and General Surgery, Provincial Specialist Hospital No. 4 in Bytom, 41-902 Bytom, Poland; slawoj1@vp.pl; 2 Department of Cardiology, Faculty of Health Sciences in Katowice, Medical University of Silesia in Katowice, Ziołowa Street 45/47, 40-635 Katowice, Poland; 3 Doctoral School, Medical University of Silesia in Katowice, 40-055 Katowice, Poland; 4 Collegium Medicum—Faculty of Medicine, WSB University, 41-300 Dąbrowa Górnicza, Poland

**Keywords:** doctors, stress, coping methods

## Abstract

**Background/Objective:** The aim of this study was to assess stress-coping patterns among Polish doctors, taking into account their degree of resilience and sociodemographic characteristics. **Methods:** This study involved 832 (100%) Polish physicians working in the Silesian Voivodeship, Poland. All respondents gave their informed and voluntary consent to participate. This study was conducted using an original questionnaire and the Resilience Measurement Scale (SPP-25) Stress Coping Inventory (Mini-COPE). **Results:** Women used the strategies: *Seeking Emotional Support* and *Seeking Instrumental Support* significantly more often than men. *Positive Re-evaluation* strategy was used more often by doctors working in surgical wards, who also declared more frequent use of psychoactive substances. Younger doctors (≤30 years) were characterized by lower mental resilience compared to other age groups and doctors with high psychological resilience were characterized by higher scores in strategies belonging to the group of active coping with stress. **Conclusions:** The examined doctors with high resilience were mostly characterized by the use of active methods of coping with stress, in contrast to doctors with low resilience. Actions should be taken to increase the awareness of healthcare system employees in the field of active stress coping techniques and their adverse effects on health.

## 1. Introduction

Stress, defined as a state of worry or mental tension caused by a difficult situation, is one of the most important issues studied in the sciences of psychology and medicine. It is a natural human reaction, prompting one to face challenges and threats in everyday life. The experience of stress is individually variable, and the way one reacts to it has a great impact on the overall well-being of the individual [1].

Coping with stress is usually defined as the conscious use of cognitive, affective, or behavioral efforts to cope effectively with externally imposed events and demands that are perceived by the individual as unpleasant or potentially harmful [2].

The process of coping with stress consists of efforts to reduce the perceived stress through a wide range of thoughts, emotions, and actions directed at both external stressors and internal demands and needs. Coping is based on two areas: the area of availability and the area of situational coping. The first includes the disposition of the person; while the second includes the conscious use of strategies to improve internal resources (e.g., self-confidence, resourcefulness, endurance, mental resilience) and coping with external demands [3].

From the perspective of medical sciences, resilience is described as the ability of individuals to grow, develop and learn in the light of traumas or challenges. In the described perspective, resilience is the individual psychological ability to return to a certain “normal” state or state of functioning after a disruptive event—coping with pressure and problems through flexibility without compromising the efficiency of the system or adapting to a new normal state in which the functioning of the system is somehow reorganized or improved in response to the disruption [4].

Among healthcare workers, fear of contagion, transmitting the infection to their family members, inadequate protective measures, and risk of medical violence imposed a burden on their mental health by stress level elevation [5]. Members of therapeutic teams present various ways of dealing with stress, taking into account, for example, their country of origin and the current epidemiological situation in its area [6].

The literature contains research results on the perception of stress and stress coping strategies by medical workers, especially during the COVID-19 pandemic, which significantly influenced the stress parameters studied among members of therapeutic teams around the world [7,8]. Single studies were also found analyzing both mental resilience and stress-coping patterns together among members of therapeutic teams in Poland [9].

An interesting area of research, therefore, seems to be the study of the connections between stress coping strategies and resilience, in a group of doctors, characterized by varying degrees of mental resilience, from the aforementioned country who are constantly exposed to stress caused by an uncertain work situation limited by legal regulations, the presence of negative internal disputes between various medical professions, significantly increasing patients demands, the negative image of the professional group often presented in the media, and the nature of the profession itself—long work hours, etc. Therefore, the aim of this study was to assess stress-coping patterns among Polish doctors, taking into account their degree of resilience and sociodemographic characteristics.

## 2. Materials and Methods

### 2.1. Sample

Before the study began, its draft was submitted for review to the Bioethics Committee of the Medical University of Silesia. Based on the decision PCN/0022/KB/287/19 of 16 December 2019, consent was obtained to conduct it, taking into account the requirements and regulations regarding the proper conduct of scientific research. Inclusion criteria were the ability to understand and answer the questions asked, as well as the willingness to participate in the study. This study involved 832 (100%) Polish physicians working in the Silesian Voivodeship, Poland. All respondents gave their informed and voluntary consent to participate.

### 2.2. Methods

This study was conducted using a questionnaire specially created for the study composed of questions on sociodemographic information of the examined physicians, including questions regarding the characteristics of their work, and two standardized questionnaires in Polish: The Resilience Measurement Scale (SPP-25) by N. Ogińska-Bulik and Z. Jurczyński and the Stress Coping Inventory (Mini-COPE) by Ch. S. Carver, in the Polish adaptation by Z. 90 Juczyński and N. Ogińska-Bulik [10,11]. Both SPP-25 and Mini-COPE were selected due to their frequent use in routine clinical practice and the ease of administration. Data were collected from September 2020 to September 2021.

### 2.3. Procedures

The Resilience Measurement Scale allows for measuring the general level of resilience and the five factors that make it up, which are as follows:

- Perseverance and determination in action;
- Openness to new experiences and a sense of humor;
- Personal competencies and tolerance of negative emotions;
- Tolerance of failures and treating life as a challenge;
- Optimistic attitude to life and the ability to mobilize in difficult situations.

The overall score of the Resilience Measurement Scale consists of the sum of the points of the five above-mentioned areas, and the interpretation is based on the assumption that a higher score corresponds to higher resilience. The obtained score can be converted according to the key into sten scores and then into resilience categories. Results of the sten score range from 1 to 4 indicating low resilience, from 5 to 6—average resilience, and from 7 to 10—high resilience of the examined people [10].

The Mini-COPE is used to assess typical ways of reacting and feeling in stressful situations. The described questionnaire consists of 28 statements, which are part of 14 strategies for coping with stress, divided into 7 groups. The first group consists of active coping strategies (Planning, Positive Re-evaluation, and active coping), while the second expresses helplessness (Substance Use, Behavioral Disengagement, and Self-Blame). The third group of behaviors consists of support-seeking strategies (Seeking Emotional and Instrumental Support), and the fourth group of avoidance behaviors (Self-distraction, Denial, and Venting). The remaining ways of coping with stress included in the described questionnaire (Turning to Religion, Acceptance, and Sense of Humor) constitute independent coping strategies [11].

The questionnaires used in the study were distributed to individual study participants in paper form using the snowball sampling method.

### 2.4. Statistical Analyses

Statistical analyses were performed using Statistica 13.3 software (Statsoft, Kraków, Poland). Qualitative data were presented taking into account the number and percentages in relation to the entire group. Quantitative data were presented taking into account descriptive statistics, including mean, standard deviation, and median. The aforementioned data were analyzed using the Shapiro–Wilk test to assess the occurrence of normal distribution. Due to the deviation of the described data from the normal distribution, nonparametric tests were used. The Mann–Whitney U test was used for comparisons between two groups, and the Kruskal–Wallis ANOVA test between more than two groups. Statistical significance was set at *p* < 0.05.

## 3. Results

### 3.1. General Characteristics of Study Group

Table 1 presents the general characteristics of the study group.

Less than 40% (322; 38.70%) of the study group were men, and the average age of the study group was 39.09 ± 10.67. Almost 80% (668; 79.33%) of the study group declared being on duty, and almost 70% (576; 69.23%) had additional employment. Less than one-third (273; 32.81%) of the study doctors were characterized by high resilience.

Table S1 (Supplementary material) presents the characteristics of the study group, taking into account the descriptive statistics of the results of the surveyed physicians in the Mini-COPE questionnaire. The highest score among the ways of coping with stress was obtained by the surveyed doctors on the following scales: *Active coping with stress* and *Planning*.

### 3.2. Characteristics of the Study Group Taking into Account Stress Coping Strategies and Sociodemographic Characteristics

Table 2 presents the characteristics of the study group, taking into account the strategies of coping with stress, as well as gender, type of ward, performing work on duty, and undertaking additional income-generating activities. Table 3 presents the characteristics of the study group, taking into account the strategies of coping with stress, the age of the respondents, and their time of work in years.

Taking into account the variable “gender”, women used the strategies: *Seeking Emotional Support* and *Seeking Instrumental Support* significantly more often than men. *Positive Re-evaluation* was used more often by doctors working in surgical wards, who also declared more frequent use of psychoactive substances. On the other hand, performing additional work significantly increased the number of points in the *active coping*. Younger respondents (25 to 30 years old) scored significantly more points in the *Seeking Emotional* and *Instrumental Support* strategies. Similar observations were made taking into account the length of service—doctors working from 1 to 10 years obtained higher point values than the other groups in the strategies *Seeking Emotional Support* and *Seeking Instrumental Support*.

### 3.3. Characteristics of the Study Group Taking into Account Mental Resilience and Sociodemographic Characteristics

The characteristics of the studied group of doctors, taking into account mental resilience and sociodemographic characteristics, are presented in Table 4.

Younger doctors (≤30 years) were characterized by lower mental resilience compared to other age groups. Considering the length of service, significant differences in the total number of points were found between those working for 1 to 10 years and those working for 21 to 30 and ≥31 years. Doctors working in surgical wards were characterized by higher point values, and, consequently, higher resilience, than their colleagues working in non-surgical wards. It was also found that doctors with additional employment compared to those working in one place obtained significantly higher point values in the *Openness to new experiences and a sense of humor subscale*, while doctors on duty compared to those not on duty in the *Optimistic attitude to life and ability to mobilize in difficult situations* subscale.

### 3.4. Characteristics of the Study Group Taking into Account Stress Coping Strategies and Mental Resilience

The average results obtained by the surveyed physicians with regard to mental resilience are presented in Table 5.

Doctors with high psychological resilience were characterized by higher scores in strategies belonging to the group of active coping with stress. They were also characterized by a higher sense of humor than the others. Doctors with low resilience, on the other hand, showed higher point values in strategies belonging to the group of helplessness and avoidance behaviors.

## 4. Discussion

According to the World Health Organization, stress has been recognized as the next epidemic of the 21st century. In the years 1983–2020, the perception of stress increased among the general public due to the increase in stressors (e.g., increase in workload, a fall in social support, uncertainty and, violence) [12]. Later on, one of the most important sources of stress not only for medical workers but also the general population was the COVID-19 pandemic, which disrupted normal work, education, healthcare, the economy, and interpersonal relationships [13,14,15]. In Poland, according to the IPSOS report, as many as 45% of Poles claim that they have experienced severe stress several times in the past year, while worldwide, this percentage is approximately 33%. Every third Pole (34%) has been so depressed many times that they felt sad or desperate almost every day for several weeks. More Polish women (39%) than men (29%) were in such a critical condition. Stress also has a negative impact on work. A total of 35% of Polish respondents admitted that they were stressed to the point that they were unable to go to work for some time. Almost every fifth respondent (19%) experienced such a state several times [16]. It has to be stated that Polish society, although slowly noticing the problem of stress and its consequences, is not tolerant enough in this area. People living with high stress struggle with problems at work and in their private lives, often unable to find help. Despite their problem, they are often forced to continue dealing with it because of the prevailing belief about the ability to cope with all problems on their own. The problem with coping with stress is particularly visible in professions that are initially burdened with a high exposure to stress, where an atmosphere conducive to dealing with it is often not created.

The presented results show how different methods of coping with stress are used by doctors in their daily life and work. The highest point values, and, consequently, the most frequently used methods of coping with stress, were methods of actively coping with it. As mentioned, active stress management should be the preferred method due to the potentially more beneficial results not only in relieving stress but also in reducing its long-term complications.

Taking into account sociodemographic data, it was shown that women used the support-seeking strategy significantly more often than men. According to the socio-ecological model, help-seeking behaviors are conditioned by individual, interpersonal, and social factors [17]. The term “help-seeking”, defined as the act of seeking a solution in order to satisfy the needs of a given person, is a process in which an individual seeks resources to satisfy the needs they have defined, such as experiences of violence or health/financial problems that are also stress factors [18].

Cultural and gender norms play a significant role in understanding the appropriateness of discussing certain issues and shaping expectations regarding different types of support. These norms also influence decisions to seek help [19].

Studies suggest that men face particular challenges in recognizing the need for professional help and actively seeking it due to the cultural and gender norms described above. Unfortunately, the results in this area are not clear. Molla S. showed that women tend to seek help less often than men because they exhibit shy behavior when seeking help, especially in the case of psychological difficulties [20]. The opposite results were obtained by Nam S. et al. who showed that women tend to seek psychological help more often than men [21]. Despite the discrepancies, it is still believed that men are less willing to seek help in any respect than women, probably because of the image of a man as strong and independent, entrenched in society and fueled by the media [22]. The observed lower willingness to actively seek help in the study group could also be caused by the profession performed, which is a priori associated with many stereotypes regarding strong character.

Our study showed that doctors working in surgical wards more often used techniques from the helplessness group, i.e., *Taking Psychoactive Substances*, despite the fact that they were characterized by higher point values in terms of resilience—they had a higher degree of resilience than their colleagues working in non-surgical wards.

The problem of substance abuse among physicians is significant. In a study conducted by Nadu T. et al. in India, among 235 respondents, 82% of physicians drank alcohol, and these were mainly surgical physicians. The rate of alcohol use was higher among physicians (82%) than in the general population (7–75%) [23]. A study conducted in Norway showed that surgeons were less likely to abstain from alcohol and more likely to engage in risky alcohol consumption than non-surgical physicians. Oreskovich M. et al., in their study, proved that factors independently associated with alcohol abuse or dependence were age (OR = 0.985; *p* < 0.0001), hours worked (OR = 0.994; *p* = 0.0094), male gender (OR = 0.597; *p* < 0.0001), being married (OR 1.296; *p* = 0.0424) or partnered (OR 1.989; *p* = 0.0003), having children (OR 0.745; *p* = 0.0049), and being in any specialty other than internal medicine (OR 1.757; *p* = 0.0060). Specialty choice was strongly associated with alcohol abuse or dependence (*p* = 0.0011). Alcohol abuse or dependence was associated with burnout (*p* < 0.0001), depression (*p* < 0.0001), suicidal ideation (*p* = 0.0004), lower quality of life (*p* < 0.0001), lower career satisfaction (*p* = 0.0036), and recent medical errors (*p* = 0.0011) [24]. Also, a study conducted in Belgium on the gender distribution in medical specialties showed that in anesthesiology, internal medicine, and gynecology/obstetrics women reported equal or even higher levels of risky alcohol drinking than men [25]. It is also worth emphasizing that during the COVID-19 pandemic, the general alcohol consumption by doctors in quarantine in Poland increased, and over 40% of them consumed alcohol more than 4 times a week due to anxiety and a sense of meaninglessness [26]. Our studies did not find any significant differences in the use of psychoactive substances as a method of coping with stress between different age groups which differentiates our study from others. In the study of medical students by Arora A. et al., it was found that a trend towards increased proportion of substance abuse was observed in the latter years of medical education [27].

It should be emphasized that the use of psychoactive substances is very dangerous and poses a threat to human health. Nowadays, opioid and narcotic use is a significant problem around the world. No data were found in the available literature on the current scale of the problem among doctors in Poland and Europe, which opens up new research possibilities in the presented scope.

Younger doctors—aged 25 to 30 years—scored significantly more points in the strategy of *Seeking Emotional* and *Instrumental Support*. In addition, younger doctors (≤30 years) were characterized by lower mental resilience compared to other age groups. The observed relationships may result from several reasons. Firstly, in the studies conducted so far, it was noticed that young people have much greater difficulties in coping with stress than older people [28]—which confirms the results of our research. Secondly, the greater intensity of stress in young doctors could result from the lack of experience or improper treatment by more experienced doctors during work—mobbing, ridicule, forcing to work. Their behavior can be attributed to previous forms of physician learning and unhealthy work patterns at the beginning of their work life. Thirdly, the observed tendency of younger physicians to actively seek help breaks the promoted image of doctors as employees who do not complain, for whom the only good is the good of the patient, ignoring their own. Studies by Graves B. et al. have shown that Seeking Instrumental and Emotional Support were the most commonly used methods of coping with stress among medical school students [29]. The activity of young medics in the area of seeking help is a positive phenomenon and should be promoted and improved by medical self-governments.

Doctors with high psychological resilience were characterized by higher scores in strategies belonging to the group of active coping with stress. They were also characterized by a higher sense of humor than the other groups. Doctors with low resilience, on the other hand, showed higher point values in strategies belonging to the group of helplessness and avoidance behaviors. Resilience provides protection against stress, among other things, in the workplace. In the discussed context, it often happens that a given individual perceives the demands of a situation as exceeding the resources available to meet them. The discussed resources may be organizational—workload or inadequate remuneration—or personal—low self-efficacy, avoidance behaviors, and distancing [30]. Exceeding the capabilities of resources causes stress and in the long term may lead to professional burnout, which is also a significant problem among Polish doctors.

In the studies conducted so far, we did not find detailed data and analyses on the influence of sociodemographic and professional factors on the ways of coping with stress among doctors, taking into account their resiliency. Therefore, the novelty of our research is based on the conducted analysis taking into account mentioned variables not yet studied properly among the population. Additionally, the study results complement and expand the available knowledge about the ways in which Polish doctors deal with stress. Moreover, our study sets a new direction for future research and is an indication for decision-makers regarding the basis for implementing programs to help doctors, especially young ones, cope with stress and prevent professional burnout.

The limitations of this study include the heterogeneity of the groups involved and the limited number of survey respondents. However, given the general reluctance of physicians to participate in research concerning them and the significant number of their professional duties, the study group allows for conclusions to be drawn. It has to also be emphasized that the study participants were not monitored for the occurrence of mental disorders such as depression or anxiety, which could have influenced the perception of stress levels. Moreover, the use of the snowball method in a selected professional group of doctors could introduce selection bias, as participants from the very beginning might share some similar characteristics/behaviors due to their profession and work-related habits.

Nevertheless, this study clearly shows how challenging it is to cope with stress, especially among younger people and those with less professional experience. Therefore, actions are needed to improve communication between young and experienced employees of the healthcare system to reduce the negative effects of stress related to the job in younger employees and to improve the mental state of older employees through conversation.

## 5. Conclusions

The examined doctors with high resilience were mostly characterized by the use of active methods of coping with stress, in contrast to doctors with low resilience. Taking into account the sociodemographic data of the examined doctors and data associated with their work, it was shown that they use different methods of coping with stress depending on, among other things: gender, working time, and type of ward. Actions should be taken to increase the awareness of healthcare system employees in the field of active stress coping techniques and of stress’ adverse effects on health.

## Figures and Tables

**Table 1:** General characteristics of the study group of doctors.

Entire Study Group (n = 832; 100%)			
Variables		Data	
		n	%
Sex	Woman	510	61.30
	Men	322	38.70
**Age [years]** M—39.09 SD—10.67 Me—35.00 Q1—31.00 Q3—46.00 Min—25.00 Max—75.00	25–30	205	24.64
	31–40	312	37.50
	41–50	176	21.15
	≥51	139	16.71
**Type of ward**	Surgical ward	274	32.93
	Non-surgical ward	558	67.07
**Working time in years** M—13.03 SD—10.68 Me—9.00 Q1—5.00 Q3—20.00 Min—1.00 Max—51.00	1–10	458	55.05
	11–20	179	21.51
	21–30	131	15.75
	≥31	64	7.69
**Work on medical duty**	Yes	668	79.33
	No	164	20.67
**Length of time on duty**	≤12 h	96	11.54
	13–24 h	430	51.68
	25–48 h	120	14.42
	>48 h	22	2.64
**Additional work**	Yes	576	69.23
	No	256	30.77
**Level of resilience**	Low	286	34.38
	Medium	264	31.73
	High	273	32.81

Abbreviations: n—number, M—mean, SD—standard deviation, Me—median, Q1—lower quartile, Q3—upper quartile, Min.—minimum value, Max.—maximum value.

**Table 2:** Characteristics of the studied group of doctors, taking into account stress coping strategies, gender, type of ward, work on duty, and undertaking additional income-generating activities.

Variables	Descriptive Statistics				Z	*p*
	M	SD	M	SD		
Sex	Women		Men			
*Seeking Emotional Support*	3.96	1.60	3.53	1.53	−4.020	<0.001
*Seeking Instrumental Support*	3.98	1.52	3.57	1.44	−4.180	<0.001
*Self-distraction*	3.44	1.42	2.92	1.42	−4.870	<0.001
*Venting*	2.91	1.32	2.43	1.31	−4.940	<0.001
*Self-Blame*	2.83	1.64	2.35	1.48	−3.720	<0.001
**Type of ward**	**Surgical ward**		**Non-surgical ward**		**Z**	***p***
*Positive Re-evaluation*	3.81	1.24	3.56	1.45	−2.452	0.014
*Substance Use*	1.01	1.44	0.79	1.32	−2.284	0.02
**Work on medical duty**	**Yes**		**No**		**Z**	***p***
*Sense of Humor*	2.15	1.40	1.80	1.30	−2.910	0.003
**Additional work**	**Yes**		**No**		**Z**	***p***
*Active Coping*	4.54	1.22	4.41	1.15	−2.036	0.04
*Seeking Emotional Support*	3.77	1.56	3.88	1.66	3.143	0.001

Abbreviations: M—mean, SD—standard deviation, Z—Mann–Whitney U test value, *p*—statistical significance.

**Table 3:** Characteristics of the studied group of doctors, taking into account stress coping strategies, age, and work experience.

Variables	Descriptive Statistics								H	*p*	Post Hoc Test:
	M	SD	M	SD	M	SD	M	SD			
Age	From 25 to 30 Years		From 31 to 40 Years		From 41 to 50 Years		≥51 Years				
*Seeking Emotional Support*	4.26	1.43	3.80	1.59	3.50	1.57	3.46	1.65	32.356	<0.001	25–30 vs. 31–40—*p* = 0.008; 25–30 vs. 41–50—*p* < 0.001; 25–30 vs. ≥51—*p* < 0.001.
*Seeking Instrumental Support*	4.28	1.35	3.89	1.46	3.52	1.53	3.37	1.59	41.422	<0.001	25–30 vs. 31–40—*p* = 0.02; 25–30 vs. 41–50—*p* < 0.001; 25–30 vs. ≥51—*p* < 0.001; 31–40 vs. 41–50—*p* = 0.04; 41–50 vs. ≥51—*p* < 0.01.
*Self-distraction*	3.51	1.44	3.32	1.27	2.91	1.53	3.06	1.60	19.981	<0.001	25–30 vs. 41–50—*p* < 0.001; 25–30 vs. ≥51—*p* = 0.03; 31–40 vs. 41–50—*p* = 0.02.
*Venting*	2.96	1.21	2.83	1.31	2.42	1.35	2.53	1.44	20.083	<0.001	25–30 vs. 41–50—*p* < 0.001; 25–30 vs. ≥51—*p* = 0.03; 31–40 vs. 41–50—*p* = 0.01.
*Self-Blame*	2.76	1.68	2.77	1.58	2.20	1.55	2.74	1.47	19.284	<0.001	25–30 vs. 41–50—*p* < 0.01; 31–40 vs. 41–50—*p* < 0.001; 51–50 vs. ≥51—*p* < 0.01.
**Working time in years**	**From 1 to 10 years**		**From 11 to 20 years**		**From 21 to 30 years**		**≥31 years**		**H**	***p***	
*Seeking Emotional Support*	4.02	1.51	3.70	1.61	3.27	1.59	3.48	1.70	28.215	<0.001	1 to 10 vs. 21 to 30—*p* < 0.001; 1 to 10 vs. ≥31—*p* = 0.04.
*Seeking Instrumental Support*	4.07	1.42	3.70	1.54	3.30	1.43	3.41	1.72	36.073	<0.001	1 to 10 vs. 11 to 20—*p* = 0.03; 1 to 10 vs. 21 to 30—*p* < 0.001; 1 to 10 vs. ≥31 *p* = 0.01.
*Self-distraction*	3.42	1.35	2.87	1.45	3.22	1.55	2.94	1.58	22.957	<0.001	1 to 10 vs. 11 to 20—*p* < 0.001.
*Denial*	1.34	1.35	1.53	1.36	1.79	1.48	1.23	1.40	14.336	<0.01	1 to 10 vs. 21 to 30—*p* < 0.01.
*Venting*	2.92	1.26	2.47	1.34	2.65	1.40	2.22	1.45	23.635	<0.001	1 to 10 vs. 11 to 20—*p* = 0.03; 1 to 10 vs. ≥31—*p* = 0.001.

Abbreviations: M—mean, SD—standard deviation, H—Kruskal–Wallis test value, *p*—statistical significance.

**Table 4:** Characteristics of the studied group of doctors, taking into account mental resilience and sociodemographic characteristics.

Tested Parameter	Mental Resilience (in Points)											
	Resilience—Overall		Perseverance and Determination in Action		Openness to New Experiences and Sense of Humor		Personal Competences for Coping and Tolerance of Negative Emotions		Tolerance for Failure and Treating Life as a Challenge		An Optimistic Attitude to Life and the Ability to Mobilize in Difficult Situations	
	M	SD	M	SD	M	SD	M	SD	M	SD	M	SD
**Sex**												
**Women**	69.27	13.53	14.15	3.57	14.64	3.39	13.30	3.64	13.69	3.93	12.41	3.65
**Men**	74.69	12.89	14.85	3.29	15.80	2.67	14.73	3.13	14.98	3.24	14.11	3.31
**Mann–Whitney U test (*p*)**	<0.01 *		<0.001 *		<0.001 *		<0.001 *		<0.001 *		<0.001 *	
**Age [years]**												
**≤30 lat**	69.38	11.53	14.31	2.99	14.88	2.67	13.56	3.03	13.79	3.40	12.50	3.19
**31–40 lat**	69.70	13.26	13.91	3.40	14.89	3.16	13.58	3.40	13.96	3.13	12.91	3.45
**41–50 lat**	73.37	15.03	14.89	3.83	15.09	3.64	14.01	4.02	14.43	4.98	13.29	4.01
**≥51 lat**	75.72	13.77	15.13	3.70	15.83	3.21	14.71	3.69	14.99	3.46	13.97	3.90
**Kruskala–Wallis test**	<0.001 *		<0.001 *		<0.001 *		<0.001 *		<0.001 *		<0.001 *	
**Post hoc analyses: age**	≤30 vs. ≥51—*p* < 0.001 * ≤30 vs. 41–50—*p* = 0.02 * 31–40 vs. 41–50—*p* = 0.03 * 31–40 vs. ≥51—*p* < 0.001 *		≤30 vs. ≥51—*p* = 0.02 * 31–40 vs. 41–50—*p* < 0.01 * 31–40 vs. ≥51—*p* < 0.001 *		≤30 vs. ≥51—*p* = 0.001 * 31–40 vs. ≥51—*p* < 0.01 *		≤30 vs. ≥51—*p* = 0.001 * 31–40 vs. ≥51—*p* < 0.01 *		≤30 vs. ≥51—*p* < 0.001 * 31–40 vs. ≥51—*p* < 0.001 *		≤30 vs. ≥51—*p* < 0.001 * 31–40 vs. ≥51—*p* < 0.01 *	
**Working time in years**												
**1–10**	69.40	12.44	14.08	3.20	14.91	2.91	13.54	3.20	13.86	3.20	12.71	3.27
**11–20**	72.88	14.20	14.72	3.67	15.12	3.37	14.09	3.68	14.41	4.75	13.31	3.82
**21–30**	74.37	15.88	14.97	4.05	15.12	4.01	14.10	4.45	14.56	4.27	13.35	4.54
**≥31**	75.55	11.80	14.92	3.37	16.20	2.32	14.95	2.83	15.16	2.35	14.31	2.95
**Kruskala–Wallis test**	<0.001 *		<0.001 *		<0.001 *		<0.001 *		<0.001 *		<0.001 *	
**Post hoc analyses: Working time in years**	1–10 vs. 21–30—*p* < 0.001 * 1–10 vs. ≥31—*p* < 0.001 *		1–10 vs. 21–30—*p* = 0.001 *		1–10 vs. ≥31—*p* < 0.01		1–10 vs. ≥31—*p* < 0.001 *		1–10 vs. 21–30—*p* < 0.001 * 1–10 vs. ≥31—*p* < 0.01 *		1–10 vs. ≥31—*p* < 0.01 *	
**Type of ward**												
**Surgical**	76.27	11.96	15.53	2.88	15.67	2.81	14.89	3.30	15.07	3.06	14.50	3.34
**Non-surgical**	70.54	13.63	14.23	3.54	14.99	3.23	13.68	3.53	14.03	3.81	12.82	3.61
**Mann–Whitney U test**	<0.001 *		<0.001 *		0.03 *		<0.001 *		<0.001 *		<0.001 *	
**Additional work**												
**Yes**	71.87	13.34	14.38	3.56	15.29	3.12	14.02	3.54	14.27	3.40	13.17	3.65
**No**	70.30	13.94	14.52	3.29	14.63	3.28	13.49	3.46	14.00	4.37	12.84	3.56
**Mann–Whitney U test**	0.239		0.628		0.006 *		0.043		0.172		0.283	
**Work on medical duty**												
**Yes**	71.77	13.54	14.44	3.52	15.15	3.18	13.92	3.51	14.29	3.81	13.21	3.61
**No**	69.90	13.46	14.34	3.32	14.85	3.20	13.60	3.56	13.80	3.36	12.50	3.61
**Mann–Whitney U test**	0.209		0.738		0.358		0.392		0.128		0.021 *	

Abbreviations: n—number, M—mean, SD—standard deviation, *p*—level of statistical significance, *—statistically significant result.

**Table 5:** Characteristics of the studied group of doctors with regard to stress coping strategies and mental resilience.

Stress Coping Strategies	Level of Resilience						Kruskal–Wallis Test		
	Low		Medium		High				Post Hoc Analysis
	M	SD	M	SD	M	SD	H	*p*	
*Active Coping*	4.02	1.07	4.48	0.93	5.16	1.09	158.985	<0.001	N vs. Ś *p* < 0.001 N vs. W *p* < 0.001 W vs. Ś *p* < 0.001
*Planning*	4.05	1.11	4.46	0.91	5.07	1.13	126.475	<0.001	N vs. Ś *p* < 0.001 N vs. W *p* < 0.001 W vs. Ś *p* < 0.001
*Positive Re-evaluation*	3.00	1.33	3.71	1.00	4.34	1.34	143.561	<0.001	N vs. Ś *p* < 0.001 N vs. W *p* < 0.001 W vs. Ś *p* < 0.001
*Acceptance*	3.63	1.04	3.81	0.95	4.21	1.45	45.323	<0.001	N vs. W *p* < 0.001 W vs. Ś *p* < 0.001
*Sense of Humor*	1.80	1.35	1.98	1.19	2.49	1.48	33.350	<0.001	N vs. W *p* < 0.001 W vs. Ś *p* = 0.001
*Turning to Religion*	2.34	1.90	2.26	1.81	2.18	2.04	1.613	0.447	-
*Seeking Emotional Support*	3.66	1.61	3.87	1.32	3.96	1.68	6.887	0.03	N vs. W *p* = 0.03
*Seeking Instrumental Support*	3.85	1.51	3.90	1.27	3.81	1.60	0.086	0.957	-
*Self-distraction*	3.25	1.37	3.21	1.36	3.33	1.52	1.458	0.482	-
*Denial*	1.71	1.46	1.54	1.32	1.11	1.32	31.627	<0.001	N vs. W *p* < 0.001 W vs. Ś *p* < 0.001
*Venting*	3.05	1.22	2.71	1.18	2.49	1.46	26.295	<0.001	N vs. Ś *p* < 0.01 N vs. W *p* < 0.001
*Substance Use*	1.11	1.53	0.81	1.30	0.68	1.20	13.837	0.001	N vs. W *p* < 0.01
*Behavioral Disengagement*	1.93	1.18	1.59	1.17	0.75	1.13	151.158	<0.001	N vs. Ś *p* < 0.01 N vs. W *p* < 0.001 W vs. Ś *p* < 0.001
*Self-Blame*	3.30	1.49	2.52	1.37	2.13	1.63	79.949	<0.001	N vs. Ś *p* < 0.001 N vs. W *p* < 0.001 W vs. Ś *p* < 0.01

Abbreviations: M—mean, SD—standard deviation, H—Kruskal–Wallis test value, *p*—statistical significance level, N—low level of resilience, W—high level of resilience, Ś—medium level of resilience.

## Data Availability

The data that support the findings of this study are available from the corresponding author, upon reasonable request.

## Supplementary Materials

The following supporting information can be downloaded at https://www.mdpi.com/article/10.3390/jcm13247539/s1, Table S1: Characteristics of the studied group of physicians, taking into account the results of the Mini-COPE questionnaire.
