# Supplementary material for: The Resilience of Polish Doctors and Their Behavioral Patterns in Coping with Work-Related Stress
Source: J Clin Med. 2024 Dec 11;13(24):7539. doi: 10.3390/jcm13247539 (PMC11728058; doi:10.3390/jcm13247539)
Supplement: Supplementary file 1 [file jcm-13-07539-s001.zip › jcm-3349404-supplementary.pdf]

**Supplementary material for article:**

***The Resilience of Polish Doctors and Their Behavioral Patterns in Coping with Work-related Stress***

**Table S1.** Characteristics of the studied group of physicians, taking into account the results of the Mini-COPE questionnaire

| Entire study group (n=832; 100%) | | | | | | | |
|---|---|---|---|---|---|---|---|
| **Analyzed scales of the Mini-COPE questionnaire** | **M** | **SD** | **Me** | **Q1** | **Q3** | **Min.** | **Max.** |
| *Active Coping* | 4,51 | 1,21 | 4,00 | 4,00 | 6,00 | 0 | 6,00 |
| *Planning* | 4,48 | 1,20 | 4,00 | 4,00 | 6,00 | 0 | 6,00 |
| *Acceptance* | 3,84 | 1,24 | 4,00 | 3,00 | 4,00 | 0 | 6,00 |
| *Seeking Instrumental Support* | 3,82 | 1,50 | 4,00 | 3,00 | 5,00 | 0 | 6,00 |
| *Seeking Emotional Support* | 3,79 | 1,58 | 4,00 | 3,00 | 5,00 | 0 | 6,00 |
| *Positive Re-evaluation* | 3,64 | 1,39 | 4,00 | 3,00 | 4,00 | 0 | 6,00 |
| *Self-distraction* | 3,24 | 1,44 | 3,00 | 2,00 | 4,00 | 0 | 6,00 |
| *Venting* | 2,73 | 1,33 | 3,00 | 2,00 | 4,00 | 0 | 6,00 |
| *Self-Blame* | 2,64 | 1,59 | 3,00 | 2,00 | 4,00 | 0 | 6,00 |
| *Turning to Religion* | 2,24 | 1,92 | 2,00 | 0,00 | 4,00 | 0 | 6,00 |
| *Sense of Humor* | 2,07 | 1,39 | 2,00 | 1,00 | 3,00 | 0 | 6,00 |
| *Denial* | 1,44 | 1,39 | 1,00 | 0,00 | 2,00 | 0 | 6,00 |
| *Behavioral Disengagement* | 1,41 | 1,26 | 1,00 | 0,00 | 2,00 | 0 | 6,00 |
| *Substance Use* | 0,87 | 1,36 | 0,00 | 0,00 | 2,00 | 0 | 6,00 |

**Abbreviations:** n—count, M—mean, SD—standard deviation, Me—median, Q1—lower quartile, Q3—upper quartile, Min.—minimum value, Max.—maximum value.

The highest score among the ways of coping with stress was obtained by the surveyed doctors on the following scales: *Active coping with stress* and *Planning*.
